# Vaginal microbiota correlations to gynecological symptoms, intimate hygiene practices, and background parameters of IVF patients: a cross-sectional study

**DOI:** 10.1007/s10815-025-03629-9

**Published:** 2025-09-01

**Authors:** Thor Haahr, Sophie Ovesen, Nina la Cour Freiesleben, Mette Brix Jensen, Helle Olesen Elbaek, Birgit Alsbjerg, Rita Laursen, Malene Tanderup, Lisbeth Prætorius, Henriette Svarre Nielsen, Anja Pinborg, Vibeke Hartvig, Thomas Roland Pedersen, Jørgen Skov Jensen, Peter Humaidan

**Affiliations:** 1 Department of Clinical Medicine, Aarhus University, Aarhus, Denmark; 2 The Fertility Clinic Skive, Skive Regional Hospital, Skive, Denmark; 3 The Fertility Clinic, Department of Obstetrics and Gynecology, Copenhagen University Hospital, Hvidovre, Denmark; 4 Department of Clinical Medicine, University of Copenhagen, Copenhagen, Denmark; 5 Fertility Clinic, Copenhagen University Hospital, Rigshospitalet 4071, Copenhagen, Denmark; 6 Stork Fertility Clinic, Copenhagen, Denmark; 7 Research Unit for Reproductive Microbiology, Statens Serum Institut, Copenhagen, Denmark; 8 Department of Gynecology and Obstetrics, Aalborg University Hospital, Aalborg, Denmark

**Keywords:** Bacterial vaginosis, Vaginal microbiota, IVF, Lifestyle factors, BMI

## Abstract

**Purpose:**

To investigate baseline parameters and vaginal intimate hygiene habits comparing IVF patients with and without vaginal dysbiosis, a cross-sectional study.

**Methods:**

Patients were grouped by the presence of vaginal dysbiosis status determined by a qPCR method (high quantities of *Gardnerella* spp. and/or *Fannyhessea vaginae*) and a 16S rRNA gene sequencing-based method (VALENCIA). Patients were asked to fill out a questionnaire alongside an interview with healthcare personnel. Prevalence ratios (PR) were computed in case of statistically significant findings between groups.

**Results:**

Among 1511 patients, the prevalence of vaginal dysbiosis by qPCR was 34%, and the prevalence of community state type IV was 32%. Prevalence of vaginal dysbiosis was higher with increasing BMI, BMI 25–30 (PR 1.23 (95% CI 1.05–1.44) and BMI > 30 (PR 1.50 (1.24–1.82). Fishy odor was reported relatively rarely, but vaginal dysbiosis was significantly more prevalent in the presence of fishy odor, PR 1.72 (95% CI 1.39–2.11). Finally, vaginal douching was highly prevalent and correlated significantly to vaginal dysbiosis, PR 1.31 (95% CI 1.11–1.53). In a sub-study, we interviewed *N* = 10 women about vaginal douching, and all of them reported the use of douching with the intention to feel clean, thus contradicting general recommendations.

**Conclusions:**

The high prevalence of vaginal dysbiosis in IVF patients is predominantly a subclinical condition which is linked to lifestyle and intimate hygiene habits.

**Supplementary Information:**

The online version contains supplementary material available at 10.1007/s10815-025-03629-9.

## Introduction

The vaginal microbiota of asymptomatic women of reproductive age is typically dominated by *Lactobacillus* species, which play a key role in maintaining a healthy vaginal environment. However, approximately 20% of women undergoing in vitro fertilization (IVF) harbor a subclinical dysbiotic vaginal microbiota, often characterized by a reduced abundance of *Lactobacillus* spp. [1]. In recent years, several studies have reported an association between genital tract dysbiosis and adverse reproductive outcomes. A recent meta-analysis demonstrated a lower clinical pregnancy rate per embryo transfer among IVF patients with vaginal dysbiosis compared to those with a *Lactobacillus*-dominated microbiota (relative risk [RR] 0.82; 95% confidence interval [CI]: 0.70–0.95; *N* = 6558 patients across 25 studies) [1]. Similarly, an optimal endometrial microbiota dominated by *Lactobacillus* spp. has also been associated with improved reproductive outcomes [2].

Vaginal microbiota composition can be classified into five major community state types (CSTs) based on 16S rRNA gene sequencing. These include CST I (*Lactobacillus crispatus*), CST II (*Lactobacillus gasseri*), CST III (*Lactobacillus iners*), CST IV (a diverse, dysbiotic group), and CST V (*Lactobacillus jensenii*) [3]. Each CST may be further subdivided into subgroups for more detailed characterization [4]. CST IV is generally considered the dysbiotic type and includes CST IV-A and IV-B, which resemble bacterial vaginosis (BV) with dominance of anaerobic bacteria such as *Gardnerella* spp. [4]. In contrast, CST IV-C is associated with aerobic vaginitis (AV), often dominated by organisms such as *Streptococcus* spp. and *Enterococcus* spp. [5].

Although the etiology of BV remains incompletely understood, current evidence suggests that BV-associated bacteria can be sexually transmitted [6]. For instance, recurrence of BV has been linked to unprotected intercourse with an untreated regular partner following antibiotic treatment [7]. Nevertheless, spontaneous resolution of BV has also been documented, adding complexity to decisions regarding if and when to initiate clinical intervention—particularly in asymptomatic IVF patients [8].

Given the potential impact of vaginal dysbiosis on fertility outcomes, it is important to identify the characteristics of IVF patients with dysbiotic microbiota. The primary aim of this study was to examine associations between baseline factors such as genital tract symptoms and intimate hygiene practices and vaginal dysbiosis. Dysbiosis was assessed either as presence or absence of aerobic vaginitis microbiota (AVM) or through classification into CSTs using the VALENCIA method based on 16S rRNA gene sequencing. AVM was considered dysbiotic based on previous findings from our group, where AVM closely resembled Nugent score-positive BV and was associated with poorer pregnancy outcomes in IVF patients [9].

In a substudy, we further explored the motivations behind women’s choices of intimate hygiene practices.

## Methods

### Study population

The present study is a cross-sectional study of 1511 IVF patients with data retrieved from a screening visit prior to IVF treatment at four IVF clinics in Denmark. All IVF patients were investigated for eligibility to the present study. Subsequently, the aim was to screen IVF patients for vaginal dysbiosis and to enroll patients into a randomized controlled trial registered in EU clinical trials register (EUDRACT 2016–002385-31). The present cohort was also prospectively registered at www.clinicaltrials.gov (NCT03420859). The study was approved prior to initiation by the Research Ethics Committee of the Central Denmark Region on December 17th 2015 (1–10-72–345-15).

The women in this study were patients at one of the four fertility clinics. All women treated at the fertility clinic pending first, second, or third IVF stimulation cycle or frozen embryo transfer cycle were asked to participate in the study. They were all approached by the permanent clinical staff upon their visits at the clinics, and an interview investigating inclusion and exclusion criteria was considered for each woman. Inclusion criteria represent the Danish population of women eligible for referral to public fertility treatment (age 18–42 years, BMI < 35).

Exclusion criteria were HIV, Hepatitis B or C, uterine malformations with surgery indication as decided by treated physician, known severe renal or hepatic impairment, CIN 2 or higher, patients treated with vitamin K antagonists (Warfarin), known or suspected hypersensitivity to clindamycin or any other antibiotic, former or current inflammatory bowel disease or any uncontrolled concomitant disease (e.g., uncontrolled diabetes, uncontrolled hypertension). Considering vaginal dysbiosis, there is no clear definition; however, in this manuscript, we use vaginal dysbiosis to cover BV-type dysbiosis (herein AVM by qPCR and CST IV-A and -B) and AV-type dysbiosis (IV-C).

### Data collection

Data were collected from December 2017 to June 2022. At inclusion, women filled in an electronic questionnaire with information on general characteristics, recent vaginal symptoms, and intimate hygiene practices. Characteristics of the study population are listed in Table 1. The questionnaire can be seen in supporting information, S1. Patients were physically in the fertility clinics while answering, and they had the opportunity to ask the research nurse for clarity. The questionnaire was first tried in piloted form and then revised by the researchers based on feedback from the patients. Moreover, an interview with a research nurse or treating physician was subsequently performed to further investigate inclusion/exclusion criteria, medical history, and to obtain information about recent antibiotic treatment. Data was directly transferred to the REDCap database hosted at Aarhus University. Apart from the possibility to ask a research nurse, correct data entry was ensured with data entry rules made in the REDCap system. Missing data was sought from the patients. If not possible, we decided not to include missing data in the analyses.

**Table 1:** Characteristics of the study population by classification as abnormal vaginal microbiota (AVM) by qPCR or community state type (CST) IV by 16S rRNA gene sequencing

	Non-AVM	AVM	*P*-value	NON-CST IV*	CST IV*	*P*-value
IVF patients	1003 (66%)	508 (34%)		521 (52%)	482 (48%)	
Age, mean (± SD)	32 (± 4)	31 (± 5)	0.51	32(± 4)	32 (± 5)	0.96
BMI, median (IQR)	24 (21–27)	25 (22–28)	< 0.01	24 (21–27)	25 (22–28)	0.03
Ethnicity			0.91			0.38
Caucasian	949 (95%)	480 (94%)		491 (94%)	452 (94%)	
Other^1^	54 (5%)	28 (6%)		30 (6%)	30 (6%)	
Relationship status			0.01			0.05
In current relationship	940 (94%)	456 (90%)		485 (93%)	434 (90%)	
Singles	55 (5%)	40 (8%)		26 (5%)	41 (9%)	
Not stated	8 (1%)	12 (2%)		10 (2%)	7 (1%)	
Gender of partner			0.52			0.37
Male partner	881 (94%)	424 (93%)		445 (92%)	408 (94%)	
Female partner	51(5%)	30 (7%)		37 (8%)	23 (5%)	
Not stated	8 (1%)	2 (0.4%)		3(1%)	3 (1%)	
Smoking			0.05			0.01
Active smoker	51 (5%)	39 (8%)		23 (4%)	41 (9%)	
No smoking	952 (95%)	469 (92%)		498 (96%)	441 (91%)	
Alcohol units per week			0.02			0.69
0–6 units	957 (95%)	470 (92%)		489 (94%)	456 (95%)	
> 7 units	46 (5%)	38 (7%)		32 (6%)	26 (5%)	
Intercourse within the past 24 h	119 (12%)	69 (14%)	0.36	56 (11%)	70 (15%)	0.09
Years trying to conceive			0.18			0.29
< 1 year	25 (2%)	18 (4%)		14 (3%)	20 (4%)	
1–2 years	338 (34%)	174 (34%)		167 (32%)	159 (33%)	
2–3 years	375 (37%)	172 (34%)		203 (39%)	161 (33%)	
> 3 years	249 (25%)	128 25%)		127 (24%)	129 (27%)	
Not stated	16 (2%)	16 (3%)		10 (2%)	13 (3%)	
Type of infertility			0.08			0.38
Primary	717 (71%)	340 (67%)		366 (70%)	326 (68%)	
Secondary	286 (29%)	168 (33%)		155 (30%)	156 (32%)	
Cause of infertility						
Tubal factor	64 (6%)	49 (10%)	0.03	39 (7%)	42 (9%)	0.49
Male factor	366 (36%)	188 (37%)	0.87	186 (36%)	183 (38%)	0.47
Ovarian	97 (10%)	38 (7%)	0.18	37 (7%)	40 (8%)	0.48
Idiopathic	440 (44%)	210 (41%)	0.35	233 (45%)	196 (41%)	0.20
Endometriosis	45 (4%)	16 (3%)	0.27	22 (4%)	15 (3%)	0.40
Others^2^	73 (7%)	59 (12%)	0.01	44 (8%)	53 (11%)	0.20
Previous chlamydia infection	286 (29%)	176 (35%)	0.02	147 (28%)	173 (36%)	0.01
Previous hydrosalpinx	16 (2%)	6 (1%)	0.65	11 (2%)	4 (1%)	0.12
Pelvic surgery	218 (22%)	125 (25%)	0.22	118 (23%)	118 (24%)	0.50
Antibiotics in previous month	25 (3%)	6 (1%)	0.12	11 (2%)	7 (1%)	0.48
Previous IVF stimulation			0.34			0.32
None	751 (75%)	392 (77%)		390 (75%)	380 (79%)	
1	144 (14%)	59 (12%)		68 (13%)	55 (11%)	
2–3	108 (11%)	57 (11%)		63 (12%)	47 (10%)	
Recent menstruation			0.93			< 0.01
Last menstruation 1 st day < 7 days	191 (19%)	101 (20%)		61 (12%)	131 (27%)	
Last menstruation 1 st day ≥ 7 days	687 (68%)	345 (68%)		387 (74%)	307 (64%)	
Not stated	125 (12%)	62 (12%)		73 (14%)	44 (9%)	
Self-swab	191 (19%)	82 (16%)	0.18	137 (26%)	88 (18%)	< 0.01
Self-reported gynecological symptoms within last month^3^						
No symptoms	521 (52%)	254 (50%)	0.48	242 (46%)	251 (52%)	0.08
Abnormal vaginal discharge	326 (33%)	176 (35%)	0.42	169 (32%)	157 (33%)	1.00
Fishy odor	36 (4%)	45 (9%)	< 0.01	22 (4%)	39 (8%)	0.01
Dyspareunia	91 (9%)	42 (8%)	0.63	36 (7%)	42 (9%)	0.29
Bleeding during intercourse	33 (3%)	16 (3%)	1.00	19 (4%)	12 (2%)	0.36
Intermittent bleeding	48 (5%)	14 (3%)	0.07	26 (5%)	15 (3%)	0.15
Fungal infection	31 (3%)	12 (2%)	0.51	15 (3%)	12 (2%)	0.85
UTI	21 (2%)	12 (2%)	0.71	12 (2%)	9 (2%)	0.67
Vaginal dryness	41 (4%)	17 (3%)	0.57	17 (3%)	14 (3%)	0.86
Abdominal pain	165 (16%)	75 (15%)	0.41	90 (17%)	69 (14%)	0.23
Dysuria	34 (3%)	15 (3%)	0.76	23 (4%)	13 (3%)	0.17

^1^Others: Black, Middle-eastern, Asian, other. ^2^Other causes of infertility: no male partner, poor ovarian reserve, uterine factor. Patients could have more than one cause of infertility. ^3^Patients could report more than one symptom. Fisher’s exact test was used for all binary variables. The continuous variables were investigated with ordinary least-squares linear regression analysis. *A total of *N* = 1003 was available for this analysis

Finally, a vaginal swab (Eswab™, Copan, Brescia, Italy) was taken either by the physician or the patient herself. Treating physicians were instructed to obtain the vaginal swab during a speculum examination in the posterior fornix for at least 5 s and to rotate the swab. Patients were instructed to insert the Eswab flocked swab at least 8 cm into the vagina and to rotate for at least 5 s. It has previously been reported that vaginal self-swabs provide similar microbiota results.

To understand why patients performed vaginal douching or used a specific intimate wash regimen, we did an additional structured interview with *N* = 30 patients at Skive Fertility Clinic, including at least *N* = 10 of each intimate hygiene group. The interview was done by a research nurse elaborating on the potential motivation behind specific washing regimes and preferred menstrual products. The patients were included after the interim where we noted a high percentage of women performing douching. Thus, we consecutively recruited the next 10 patients who answered yes to douching, intimate soap, and normal soap. The same patient was allowed to participate both in the douching and in the intimate wash interview. The interview was done by a research nurse either by phone or in person. Upon this interview, the results were documented as reported in Fig. 3.

The substudy interview was conducted by the research nurse either at the clinic or by phone. We developed binary and categorical answers for some questions and left space for more elaboration/comments which we then aggregated at the time of writing the present manuscript.

The questionnaires for both the primary study and the substudy is included as PDFs in supplemental information.

### Sample processing and analysis

The vaginal swabs were placed in the Eswab tube and sent at room temperature for analysis within 7 days at Statens Serum Institute, Denmark, using quantitative PCRs for the detection of *Fannyhessea (F.) vaginae* and *Gardnerella* spp. DNA from 100 µL of the vaginal screening sample was released boiling in 300 µL Chelex resin slurry as previously described [10]. Quantitative (q)PCRs detecting *Gardnerella* spp. and *Fannyhessea (F.) vaginae* (previously *Atopobium vaginae*) were performed as previously described [11]. Abnormal vaginal microbiota (AVM) was diagnosed in samples with more than 5.7 × 10^7^ and/or 5.7 × 10^6^ copies/ml for *Gardnerella* spp. and *F. vaginae*, respectively.

Moreover, at the end of the study, the entire batch of vaginal samples was re-purified and underwent 16S rRNA gene sequencing according to a protocol described in detail previously [12]. The bioinformatics pipeline was also described in detail. Unfortunately, at this time point, only 1254 unique samples were available; the others were not to be found. Broad-range PCR targeting of the V3–V4 hypervariable region of the 16S rRNA gene was performed with primers as described in Golob et al. [13]. Using a cut-off of 1500 reads, a total of 1003 samples were in the CST analysis. In brief, the CSTs described herein were computed by the VALENCIA classifier as published by France et al. [4]. The heatmap computed in this manuscript was made using R version 4.2.1 [14].

### Statistics

The power calculation was based on the number needed to screen to randomize 333 AVM positive IVF patients in the RCT previously mentioned [15]. Binary variables are presented as total number and percentage, whereas continuous variables are presented as mean with standard deviation or median and interquartile range based on normality and equal variances. Fisher’s exact test, ANOVA, or Kruskal–Wallis were used as appropriate; all *P*-values are two-sided, and a level less than 0.05 was considered statistically significant. In case of statistical significance, the crude prevalence ratio (PR) was computed using binary regression function with loglink. The adjusted analysis was adjusted for BMI, relationship status, smoking, and alcohol as binary variables. The statistical analyses were performed using Stata version SE 18.0 (StataCorp, College Station TX).

## Results

### Demographics

**Fig. 1:**
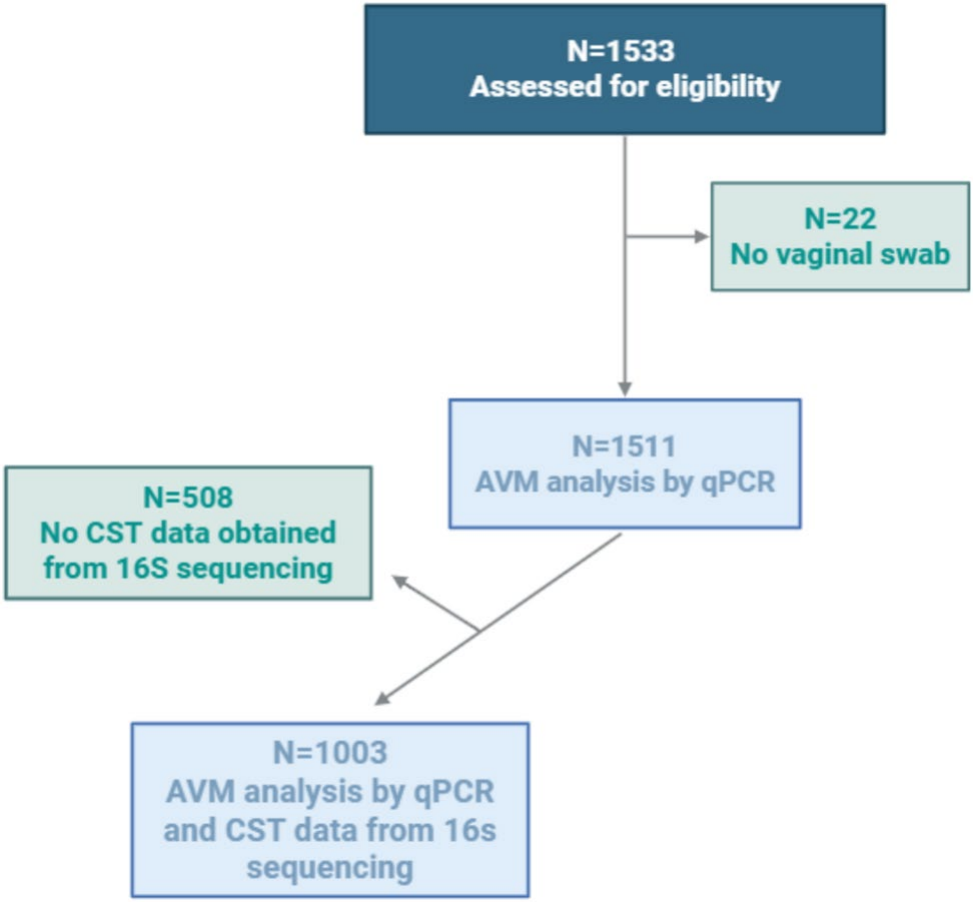
Flowchart. Flowchart depicting the recruitment of participants. Community state types (CST), abnormal vaginal microbiota (AVM)

A total of 1533 women were screened (Fig. 1); however, 22 (1%) patients did not have a vaginal swab diagnosis registered. Among the 1511 participants with a vaginal swab diagnosis, 508 (34%) women were diagnosed with AVM.

There were no statistical differences in age, ethnicity, recent intercourse (< 24 h) or recent menstruation (< 7 days) when considering the prevalence of AVM, see Table 1. When compared to patients with BMI < 25, there was a significantly higher prevalence of AVM among women with BMI 25–30 (PR 1.23 (95% CI 1.05–1.44) and BMI > 30 (PR 1.50 (1.24–1.82). Moreover, IVF patients consuming seven or more units of alcohol per week were significantly more likely to have AVM, PR 1.37 (95% CI 1.07–1.77) when compared to women consuming less than seven units per week. Among active smokers (6%) significantly more women had AVM when compared to non-smokers, PR 1.31 (95% CI 1.02–1.68). Tubal factor infertility was significantly associated with a higher prevalence of AVM, PR 1.32 (95% CI 1.06–1.65) when compared to all other causes of infertility. In the infertility group designated “other” there were also significantly more AVM-positive patients, PR 1.37 (95% CI 1.12–1.68). This group comprised a high proportion of women without a male partner and, thus, also women having sex with women (WSW), known to have a high prevalence of BV [16]. Women who have previously had a self-reported chlamydia infection were significantly more likely to have AVM, PR 1.20 (95% CI 1.04–1.39) compared to women who never had *Chlamydia*. A total of 736 (49%) IVF patients reported gynecological symptoms within the preceding month, but only fishy odor was significantly associated with AVM, PR 1.72 (95% CI 1.39–2.11).

For comparison, we also investigated the study population characteristics according to grouping by CST. Data on CST was available from 66% (1003/1511) of the patients. Significantly more vaginal swabs of the non-AVM vaginal microbiota group (496/1003) were missing compared to the AVM group (12/508), *P* < 0.01. CST IV prevalence was generally associated with the same characteristics as AVM, although we noticed a few differences, namely, tubal factor infertility and recent menstruation. As CST IV is a grouping of both BV-type and AV-type vaginal dysbiosis, we compared the prevalences of CST IV-A + B (BV type) and CST IV-C (AV-type) differentially in case of a statistically significant difference in the abovementioned variable. Patients with tubal factor infertility were significantly more likely to have CST IV-A + B, PR 1.36 (95% CI 1.04–1.77) compared to all other CSTs. In contrast, this was not seen for CST IV-C, PR 0.55 (95% CI 0.26–1.13) when compared to all other CSTs. Moreover, CST IV-C was significantly correlated with recent menstruation < 7 days, PR 3.23 (95% CI 2.43–4.30) compared to all other CSTs. This was not seen for CST-IVA + B, PR 1.00 (95% CI 0.97–1.04).

### Community state types (CST)

**Fig. 2:**
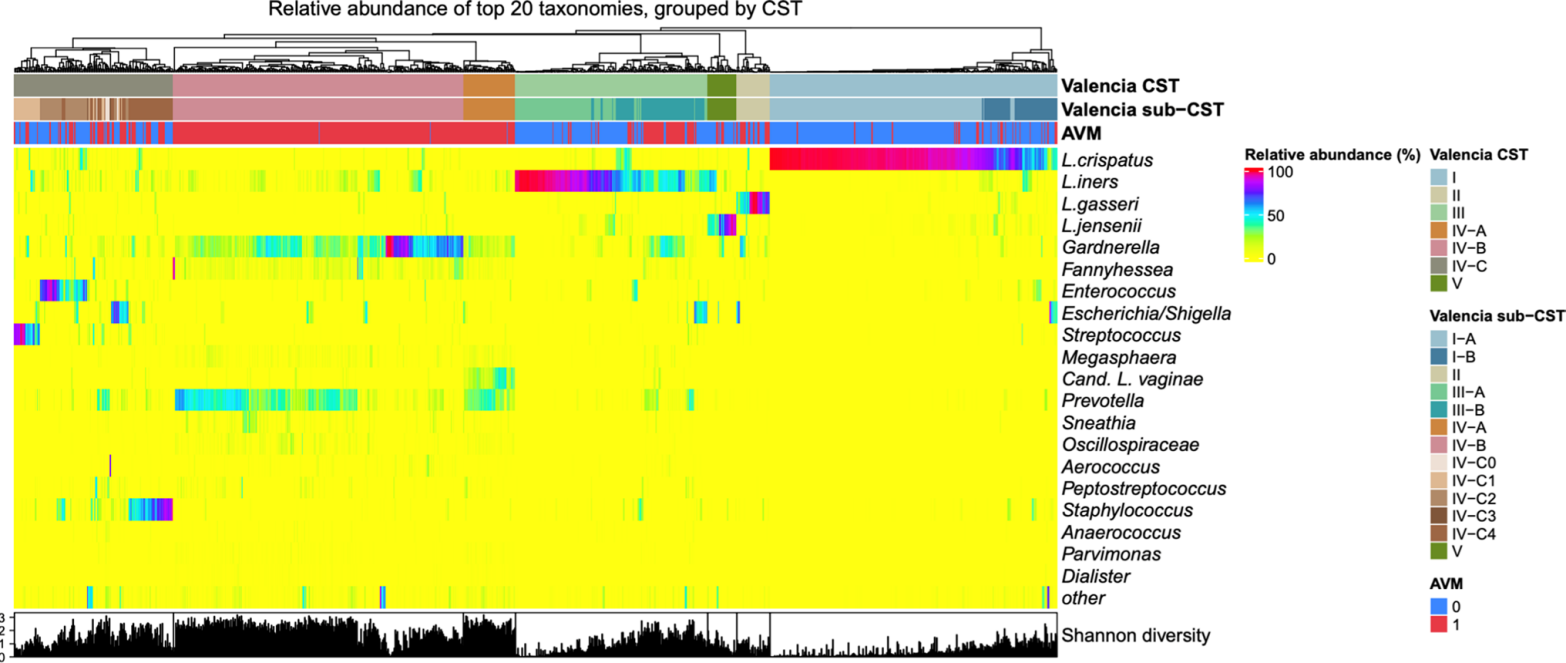
Heatmap based on 16 s rRNA gene sequencing. Heatmap showing the VALENCIA groups of community state types (CST) in the top horizontal bars. Next horizontal bar is the diagnosis of abnormal vaginal microbiota (AVM) by qPCR (blue = NOT AVM, red = AVM). Then, the heatmap is based on collapsed ASVs of top 20 taxonomies in the dataset. Below, the Shannon diversity index is presented in vertical bars

The prevalence of AVM among CST groups can be seen in Table 2. The prevalence of AVM was highest in CST IV-A (98% [95%CI 94–100%]) and CST IV-B (99% [95%CI 98–100%]) whereas the prevalence of AVM was lowest in CST I (11% [95%CI 7–14%]). When comparing CST by the VALENCIA classifier to AVM diagnosis, the largest proportion of patients with AVM were grouped in CST IV (77% [95%CI 73%–81%]). Previously, we found that patients with a Shannon index > 0.93 had a poor reproductive outcome^12^; thus, we highlight that 70% [95%CI 66–73%] of IVF patients with a Shannon index > 0.93 had AVM. An in-depth graphical presentation of the above can be seen in the heatmap in Fig. 2.

**Table 2:** Distribution and prevalence of abnormal vaginal microbiota according to Community state types among IVF patients. The table shows the AVM prevalence within the different CSTs in the third vertical line

	Non-AVM, *N* = 507	AVM, *N* = 496	AVM prevalence [95%CI]
CST I	247 (49%)	29 (6%)	11% [7–14%]
CST II	15 (3%)	17 (3%)	53% [36–70%]
CST III	124 (24%)	61 (12%)	33% [26–40%]
CST IV-A	1 (0.2%)	49 (10%)	98% [94–100%]
CST IV-B	3 (1%)	276 (56%)	99% [98–100%]
CST IV-C	96 (19%)	57 (11%)	37% [30–45%]
CST V	21 (4%)	7 (1%)	25% [9–41%]
Shannon index > 0.93	189 (37%)	439 (89%)	70% [66–73%]

### Intimate hygiene and menstrual habits

A total of 34% (513/1511) of the women used water only for their intimate washing regimen. When using soap, more women tended to use intimate soap with low pH (37%, 563/1511) compared to the use of normal soap (26%, 392/1511). The present study reports a higher rate of AVM when using intimate soap compared to using just water (Table 3). Likewise, vaginal douching was also significantly associated with AVM, PR 1.31 (95% CI 1.11–1.53).

**Table 3:** Intimate hygiene and menstrual habits according to AVM or CST IV

	Non-AVM, *N* = 1003	AVM, *N* = 510	AVM PR [95%CI]	AVM aPR [95%CI	CST IV PR [95%CI]	CST IV aPR [95%CI]
Wash regimen*,**						
Only water	356 (35%)	157 (31%)	Ref	Ref	Ref	Ref
Normal soap	266 (27%)	126 (25%)	1.05 [0.86–1.28]	1.05 [0.86–1.27]	1.05 [0.88–1.25]	1.08 [0.91–1.29]
Intimate soap	352 (35%)	211 (42%)	1.22 [1.03–1.45]	1.22 [1.03–1.44]	1.25 [1.07–1.46]	1.23 [1.05–1.44]
Other/missing	29 (3%)	14 (3%)	1.07 [0.68–1.67]	0.49 [0.20–1.21]	0.84 [0.52–1.37]	0.70 [0.34–1.45]
Use of probiotics^1^	20 (2%)	11 (2%)	1.05 [0.65–1.70]	1.09 [0.67–1.76]	0.89 [0.54–1.46]	0.94 [0.59–1.50]
Douching	170 (17%)	121 (24%)	1.31 [1.11–1.53]	1.29 [1.10–1.52]	1.22 [1.05–1.41]	1.22 [1.06–1.42]
Only water	152 (15%)	112 (22%)	Ref	Ref	Ref	Ref
Other^2^	18 (2%)	9 (2%)	0.79 [0.45–1.36]	0.81 [0.46–1.40]	0.78 [0.47–1.29]	0.79 [0.48–1.29]
Menstrual habits^3^	*N* = 766	*N* = 387				
Pads	298 (39%)	164 (42%)	Ref	Ref	Ref	Ref
Tampons	182 (24%)	105 (27%)	1.03 [0.85–1.25]	1.01 [0.83–1.23]	1.09 [0.91–1.30]	1.06 [0.89–1.27]
Pads/tampons	163 (21%)	78 (20%)	0.91 [0.73–1.14]	0.92 [0.74–1.15]	1.06 [0.87–1.28]	1.06 [0.87–1.27]
Cup	117 (15%)	38 (10%)	0.69 [0.51–0.93]	0.72 [0.53–0.97]	0.97 [0.77–1.23]	0.98 [0.77–1.25]
Other	6 (1%)	2 (1%)	0.70 [0.21–2.35]	0.87 [0.27–2.76]	0.09 [0.21–2.72]	*

^*^For patients who answered both water and soap, these were regarded as soap users. If reporting to use both regular and intimate soap, they were regarded as regular soap users. ^1^Regular use of over-the-counter probiotics compared to non-users. ^2^Including vinegar (*N* = 3), over-the-counter products from pharmacy (*N* = 10), unknown product (*N* = 13). ^3^These questions were not asked until March 2019 and thus, the total number *N* is 1153 for this variable. The percentages are relative to this number. PR = Prevalence ratio between index group and non-index group

^**^Too few observations to allow adjusted estimates

*APR* adjusted prevalence ratios. AVM adjusted for BMI, relationship status, smoking, alcohol. CST IV adjusted for BMI, relationship, smoking

The use of pads and tampons was not significantly associated with AVM or CST IV status. Using a menstrual cup was associated with a significantly lower prevalence of AVM, PR 0.69, (95% CI 0.51–0.93) when compared to using pads for menstrual hygiene.

### Subpopulation interview

**Fig. 3:**
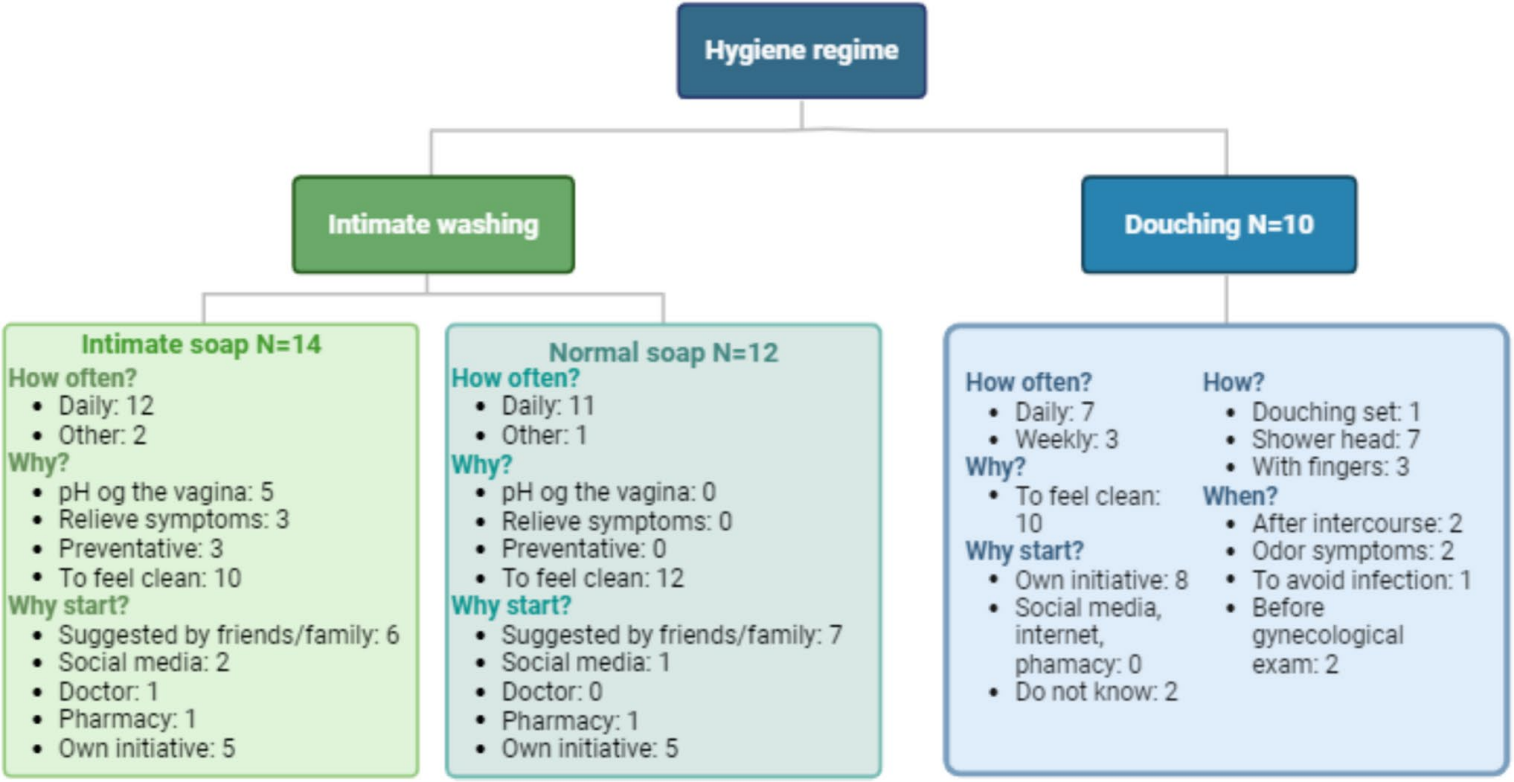
Subpopulation interview. The figure illustrates the answers of the women in the subpopulation interview about intimate hygiene regimes. More than one answer allowed per patient

As part of a further interest as to why women use a given intimate wash regimen, a subpopulation of 26 women was asked by interview with a research nurse why they had chosen the specific wash regimen. The answers are illustrated in Fig. 3. Soap was often used daily and with the purpose to feel clean. Likewise, vaginal douching was predominantly performed daily and with the purpose to feel clean.

## Discussion

### Main findings

The present study reports a relatively high prevalence of vaginal dysbiosis in IVF patients defined either as AVM (34%) or CST-IV (48%). We corroborate the findings of previous studies in non-IVF patients that also in IVF patients vaginal dysbiosis is associated with higher BMI, alcohol consumption, and smoking. Fishy odor was reported relatively rarely but was significantly more common in patients with AVM or CST IV when compared to patients not reporting this symptom. Likewise, tubal factor infertility was significantly associated with prevalent AVM or CST IV-A and IV-B. AVM was previously defined as a means to have a reliable and fast turn-around method to identify IVF patients with vaginal dysbiosis associated with a poor reproductive outcome [9]. We confirm previous results that the AVM diagnosis correlates well with BV-type microbiota CST IV-A and IV-B; albeit, more rarely, AVM can also be found outside of these CSTs. As could be expected, the dominating CST among the non-AVM positive patients was CST I (41%). In contrast, women with AVM predominantly had CST-IV-B dysbiosis (56%) followed by CST III (12%), CST IV-A (10%) and CST IV-C (11%).

Despite no guidelines recommending the use of soaps for intimate washing, we report that 63% of the patients used soaps as part of their intimate washing regimen. In a small sub-cohort undergoing interview, IVF patients reported that they used the particular washing regimen daily in order to feel clean. Moreover, even though clinical guidelines recommend against vaginal douching, this was done by 19% of the patients, and douching was significantly associated with vaginal dysbiosis, as previously reported for non-IVF patients [17] Finally, the use of low pH soap was significantly more common in IVF patients with vaginal dysbiosis, whereas the use of a menstrual cup was reported significantly more often by patients not having AVM.

### Strengths and limitations

The present study is a large cross-sectional study including 1533 IVF patients screened for AVM prior to an RCT. Thus, the present study by design cannot infer causal inference but only report associations between vaginal dysbiosis and the variables described. To our knowledge, this is the largest study in IVF patients to describe such associations, including a description of intimate health habits. The study population consisted of primarily Caucasian IVF patients, limiting the generalizability to other populations.

In March 2019 questions on the use of menstrual products were added, with a total of 1153 women completing the questionnaire (Fig. 1). This should not give cause for selection bias since the IVF patients were included sequentially by the same inclusion criteria, only at a later point in time. We cannot exclude a recall bias of the self-reported gynecological symptoms. Vaginal microbiota was investigated by means of both a qPCR method and a 16S rRNA gene sequencing-based method. Longitudinal studies have shown that the vaginal microbiota varies over the menstrual cycle [18]. A limitation of our study is that patients were screened at different days in the menstrual cycle and only on one day each.

### Interpretation

In a recent meta-analysis of observational studies, the prevalence of vaginal dysbiosis was 19% (1271/6835, 95% CI 18–20%) [19]. In the present study, we report a significantly higher prevalence of vaginal dysbiosis defined by either AVM (34%) or CST-IV (48%). The AVM prevalence reported herein is relatively similar to our previous pilot study in which we reported an AVM prevalence of 28% (*N* = 36/130) [9]. However, in that same cohort, the CST IV prevalence was only 17% (*N* = 20/120), which is significantly different in the present larger cohort. It is not straightforward to explain this difference, although we found that patients with CST IV-C seem to be highly correlated with recent menstruation whereas AVM, CST IV-A, and IV-B do not. The present larger study may have sampled more women within seven days of menstruation compared to our pilot study, which could in part explain a difference in CST IV prevalence. Importantly, it is reported herein that an AVM diagnosis, which was the primary diagnostic marker for intervention in a subsequent RCT, was not related to recent menstruation, which is in line with the basic idea behind the qPCR method, targeting a high quantity of BV-type bacteria prior to the IVF embryo transfer procedure.

Lifestyle and hygiene habits may affect the vaginal microbiota; in particular, smoking [20, 21], increased BMI [21], and low dietary fiber intake [22] are all associated with vaginal dysbiosis. Herein, we confirm these findings; albeit, we do not have information on dietary habits, which could be interesting to investigate further. As the normal vaginal microbiota acts as a defense system against ascending infections, a serious implication of vaginal dysbiosis may be an increased risk of acquiring other genital tract infections such as Chlamydia, Gonorrhea, HSV, HPV [23], and possibly also *Mycoplasma genitalium*. Consequently, these infections may lead to complications such as pelvic inflammatory disease and tubal factor infertility (TFI). In the present study, IVF patients who previously had a chlamydia infection were significantly more likely to have AVM. Moreover, and perhaps consequently, TFI was also significantly associated with a higher prevalence of AVM and CST IV-A + B. It is interesting to speculate if the higher AVM prevalence in women with TFI or previous chlamydia is the result or the cause. If a treated infection can lead to persistent changes in the microbiota, this may justify studies on the mechanism behind this.

In line with the present results, douching has repeatedly been associated with vaginal dysbiosis [17, 21]. At the clinic, in line with international recommendations, we recommend that douching should not be practiced. It has been a generally held belief that douching is not used among women in Denmark today. Thus, we were surprised to find that 19% of IVF patients practice vaginal douching. It appears that there is an incentive to achieve a feeling of cleanliness, as reflected in the answers from the small subpopulation interview. Thus controversial, as one could argue this practice may result in the exact opposite of what is intended by these women: a disruption in dominance of L*actobacillus* spp. and a higher prevalence of vaginal dysbiosis. We do not know how common douching is among Danish women in general, compared to this group of IVF patients. It is interesting to speculate whether this group of women is more motivated to feel clean due to a fertility desire while undergoing fertility treatment with frequent gynecologic exams and procedures.

The use of intimate soap was significantly associated with AVM/CST IV-A + -B in IVF patients. Despite low pH, intimate soap might disrupt the balance of the vaginal microbiota, albeit interpretation must be done with caution as the present study cannot distinguish if the use of a product is due to symptoms such as smell, itch, or change in discharge—or if the product induces dysbiosis. Perhaps the use of soaps is higher due to an attempt to alleviate symptoms of vaginal dysbiosis.

In the entire study population, 37% of the women used intimate soap, but of women who had reported fishy odor, a total of 55% used intimate soap. Considering self-reported vaginal gynecological symptoms, only vaginal fishy odor symptoms were significantly more common among patients with AVM compared to patients without AVM. Moreover, IVF doctors did not document significantly more BV symptoms in the AVM group (data not shown).

Conflicting results have been reported on the use of menstrual products. Our results showed no significant correlation between vaginal microbiota and the use of tampons or pads. In contrast, IVF patients using a menstrual cup were significantly less likely to have AVM. In a recent RCT, the use of a menstrual cup in Kenyan women is shown to significantly reduce BV and improve relative abundance of *L. crispatus* [24]. Future studies may enable optimized evidence-based recommendations on intimate health hygiene health, which is evidently needed as women prioritize the importance of feeling clean.

## Conclusion

The high prevalence of vaginal dysbiosis in IVF patients is predominantly a subclinical condition which is associated with lifestyle and intimate hygiene habits. It could be speculated that an alternative non-causal mechanism explaining the association between vaginal dysbiosis and the reproductive outcome might be that IVF patients with vaginal dysbiosis have a phenotype or specific lifestyle which, apart from predisposing the patients to vaginal dysbiosis, also makes them more prone to adverse reproductive outcomes.

## Supplementary Information

Below is the link to the electronic supplementary material.

Supplementary file1 (PDF 148 KB)

Supplementary file2 (PDF 5 KB)

Supplementary file3 (DOCX 17 KB)

## Data Availability

Dataset may be available upon request.
