# Supplementary material for: Vaginal microbiota correlations to gynecological symptoms, intimate hygiene practices, and background parameters of IVF patients: a cross-sectional study
Source: J Assist Reprod Genet. 2025 Sep 1;42(10):3443–52. doi: 10.1007/s10815-025-03629-9 (PMC12602798; doi:10.1007/s10815-025-03629-9)
Supplement: Supplementary file 1 — Supplementary file1 (PDF 148 KB) [file 10815_2025_3629_MOESM1_ESM.pdf]

Intimate Hygiene Questions – Follow-up
Record ID

Intimate soap, regular soap, both or water

☐ Intimate soap

☐ Regular soap

☐ Both

☐ Water

For patients using both intimate soap and regular soap: When do they use intimate soap? (Check all that apply)

☐ During menstruation

☐ After menstruation

☐ After intercourse

☐ When experiencing odor

☐ When experiencing discharge

☐ When experiencing itching

☐ Other

If other, please specify:

For patients using both intimate soap and regular soap: When do they use regular soap?

☐ During menstruation

☐ After menstruation

☐ After intercourse

☐ When experiencing odor

☐ When experiencing discharge

☐ When experiencing itching

☐ Other

If other, please specify:

Do you use intimate soap/regular soap daily for intimate hygiene throughout your entire cycle?

☐ Yes

☐ No

If no, when do you use intimate soap/regular soap?

☐ During menstruation

☐ After menstruation

☐ After intercourse

☐ When experiencing odor

☐ When experiencing discharge

☐ When experiencing itching

☐ Other

If other, please specify:

Why do you use intimate soap/regular soap? What is the reason?

☐ To maintain the vaginal pH

☐ To relieve symptoms such as itching, odor, and discharge

☐ To prevent symptoms such as itching, odor, and discharge

☐ To feel clean

☐ Other

If other, please specify:

Why do you only use water? What is the reason?

☐ To maintain the vaginal pH

☐ To relieve symptoms such as itching, odor, and discharge

☐ To prevent symptoms such as itching, odor, and discharge

☐ To feel clean

☐ Other

If other, please specify:

How many days per cycle do you typically use intimate soap/regular soap?

☐ 1 day

☐ 1–3 days

☐ 3–5 days

☐ 7 days

☐ More than 7 days

☐ Only when I have symptoms (i.e. not every cycle)

Where did you hear about using intimate/regular soap for intimate hygiene?

☐ Acquaintances (family, friends)

☐ Internet

☐ Social media

☐ Doctor

☐ Pharmacy

☐ Have ‘always’ done it / thought of it myself

☐ Other

If other, please specify:

Do you use vaginal products that contain bacteria (e.g. Vivag, Lactocare, etc.)?

☐ Yes

☐ No

Do you use bacterial products daily for intimate hygiene throughout your entire cycle?

☐ Yes

☐ No

If no, when do you use bacterial products?

☐ During menstruation

☐ After menstruation

☐ After intercourse

☐ When experiencing odor

☐ When experiencing discharge

☐ When experiencing itching

☐ Other

If other, please specify:

Why do you use bacterial products? What is the reason?

☐ To maintain the vaginal pH

☐ To relieve symptoms such as itching, odor, and discharge

☐ To prevent symptoms such as itching, odor, and discharge

☐ To feel clean

☐ Other

If other, please specify:

How many days per cycle do you use bacterial products?

☐ 1 day

☐ 1–3 days

☐ 3–5 days

☐ 7 days

☐ More than 7 days

☐ Only when I have symptoms (i.e. not every cycle)

Where did you hear about using vaginal bacterial products?

☐ Acquaintances (family, friends)

☐ Internet

☐ Social media

☐ Doctor

☐ Pharmacy

☐ Have 'always' done it / thought of it myself

☐ Other

If other, please specify:

Do you rinse your vagina internally (not the outer labia, but inside)?

☐ Yes

☐ No

If you have changed your douching habits – why? Please write a short explanation:

How do you rinse your vagina internally?

☐ With a rinse set (similar to an ear syringe)

☐ With the shower hose without the showerhead

☐ With fingers

☐ With steam (vaginal steaming)

☐ Other

If other, please specify:

How often do you rinse your vagina?

☐ Daily

☐ Weekly

☐ Monthly

☐ Less often

Why/when do you rinse your vagina? (You may choose multiple)

☐ To keep the vagina clean

☐ During menstruation

☐ After intercourse

☐ When experiencing odor

☐ To avoid infection

☐ To feel clean before a doctor visit

☐ Other

If other, please specify:

Where did you hear about internal vaginal rinsing (douching)?

☐ Acquaintances (family, friends)

☐ Internet

☐ Social media

☐ Doctor

☐ Pharmacy

☐ Have ‘always’ done it / thought of it myself

☐ Other

If other, please specify:

Which types of menstrual products do you use during your period? (You may choose multiple)

☐ Pad

☐ Tampon (regular)

☐ Panty liner

☐ Menstrual cup

☐ Soft tampons (sponge tampons)

☐ Natural sponge

☐ Other

If other, please specify:

Do you use menstrual products when you are not menstruating?

☐ Yes

☐ No

If yes, which ones?

☐ Pad

☐ Tampon (regular)

☐ Panty liner

☐ Menstrual cup

☐ Soft tampons (sponge tampons)

☐ Natural sponge

Why?

☐ Discharge

☐ Spotting

☐ To protect underwear

☐ Other

If other, please specify:

What do you think about being asked about your intimate hygiene in this way?

☐ It feels intimidating/uncomfortable

☐ It is liberating/a good idea to ask about it

☐ It is okay/I don't mind

☐ Don't know
