# Supplementary material for: Vaginal microbiota correlations to gynecological symptoms, intimate hygiene practices, and background parameters of IVF patients: a cross-sectional study
Source: J Assist Reprod Genet. 2025 Sep 1;42(10):3443–52. doi: 10.1007/s10815-025-03629-9 (PMC12602798; doi:10.1007/s10815-025-03629-9)
Supplement: Supplementary file 2 — Supplementary file2 (PDF 5 KB) [file 10815_2025_3629_MOESM2_ESM.pdf]

Questionnaire - FEMALE

Dear Patient,

You are participating in the study:

"The microbiome of infertile couples and its effect on their reproductive outcomes"

Would you kindly complete the attached questionnaire?

Best regards,

Principal Investigator,

Professor Peter Humaidan

Relationship Status

- Are you currently in a committed relationship?
  - Yes
  - Single

- If yes, is your partner:
  - Male
  - Female

Personal Information

- Age (in years): ________________
- Weight (in kg): ________________ (e.g., 70 - do not write letters)
- Height (in cm): ________________ (e.g., 170 - do not write letters)

- BMI: _______________

Fertility History

- How long have you/you and your partner been trying to conceive?
  (in years): _______________

- Previous pregnancies?
  - Yes
  - No

- Number of previous pregnancies
  - 1 2 3 4 5 More

What happened to the pregnancy?
(If multiple pregnancies, answer for first one, and tick "+other pregnancy" for additional ones. You can select more than one.)

- Positive urine test
- Pregnancy loss before 16 weeks
- Pregnancy loss after 16 weeks
- Live birth
- +Other pregnancy
- Induced abortion

(Repeated multiple times to cover up to 5 pregnancies)

Previous Fertility Treatments

- Number of previous IUI/insemination treatments
  - 0 1 2 3 4 5 More

- Number of previous IVF treatments
  - 0 1 2 3 4 5 6 More

Medical History

- Previous Chlamydia infection?
  - Yes
  - No

- Number of Chlamydia infections: ________________

- Previous Gonorrhoea?
  - Yes
  - No

- Pelvic surgery?
  - Yes
  - No

- What was the surgery for?

  ____

- Do you have endometriosis?

  (If unsure, answer "No")
  - Yes
  - No

Lifestyle

- Do you smoke?
  - Yes
  - No

- Number of cigarettes per day: _______________

- How many alcoholic drinks do you consume per week?

  ____

- Have you taken antibiotics in the past month?
  - Yes
  - No

Genital Symptoms (within the last month)

Have you experienced any of the following? (You may tick more than one)

- No symptoms
- Vaginal discharge with fishy smell
- Pain during intercourse
- Bleeding during intercourse
- Bleeding unrelated to intercourse or menstruation
- Yeast infection
- Urinary tract infection or cystitis
- Vaginal dryness
- Abdominal pain
- Burning sensation when urinating

Did the symptoms bother you?

- No bother
- Mild bother (no special precautions taken)
- Symptoms required action
- Severe symptoms requiring medical attention

Other Symptoms (past month)

Have you experienced any of the following? (You may tick more than one)

- None of the below
- Headache
- Diarrhea
- Extreme fatigue
- Vomiting
- Muscle/joint pain

Vaginal Hygiene

Which of the following do you use for intimate/vaginal hygiene? (Tick all that apply)

- Intimate wash
- Regular soap
- Vaginal products with bacteria
- Other
- Water only, no soap

Which product(s) do you use?

____

Which "other" product do you use?

____

Do you rinse inside the vagina? (Not external labia - but inside)

- Yes
- No

Do you use any of the following rinsing agents? (Tick all that apply)

- Water only
- Water with vinegar
- Water with natural products (from pharmacy/drugstore)
- Other

Menstruation

- Date of first day of your last period: _______________

What do you use during menstruation? (Choose what suits you best)

- Tampons
- Pads
- Sometimes pads, sometimes tampons, depending on what suits me
- Menstrual cup
- Other: _______________

Sexual Activity

- Have you had sexual intercourse in the past 24 hours?
  - Yes
  - No

- With or without condom?
  - With condom
  - Without condom
