# Supplementary material for: Vaginal microbiota correlations to gynecological symptoms, intimate hygiene practices, and background parameters of IVF patients: a cross-sectional study
Source: J Assist Reprod Genet. 2025 Sep 1;42(10):3443–52. doi: 10.1007/s10815-025-03629-9 (PMC12602798; doi:10.1007/s10815-025-03629-9)
Supplement: Supplementary file 3 — Supplementary file3 (DOCX 17 KB) [file 10815_2025_3629_MOESM3_ESM.docx]

Supplementary

**Supplementary table 1: demographics of the subpopulation**

| Patients | N=30 |
| --- | --- |
| Age, mean (±SD) | 32.1 (±4.6) |
| BMI, median (IQR) | 24.0 (22.6-29.0) |
| Ethnicity |  |
| Caucasian | 29 (97%) |
| Other^1^ | 1 (3%) |
| Relationship status |  |
| In current relationship | 30 (100%) |
| Gender of partner |  |
| Male partner | 30 (100%) |
| Smoking |  |
| Active smoker | 1 (3%) |
| No smoking | 29 (97%) |
| Alcohol units per week* |  |
| 0-6 units | 25 (93%) |
| >6 units | 2 (7%) |
| Years trying to conceive |  |
| <1 year | 9 (31%) |
| 1-2 years | 12 (41%) |
| 2-3 years | 4 (14%) |
| >3 years | 4 (14%) |
| Not stated | 1 (3%) |
| Type of infertility |  |
| Primary | 15 (50%) |
| Secondary | 15 (50%) |
| Cause of infertility |  |
| Tubal factor | 7 (23%) |
| Male factor | 8 (27%) |
| Ovarian | 2 (7%) |
| Idiopathic | 12 (40%) |
| Endometriosis | 2 (7%) |
| Previous chlamydia infection | 10 (33%) |
| Previous hydrosalpinx | 2 (7%) |
| Pelvic surgery | 15 (50%) |
| Antibiotics in previous month | 0 |
| Previous IVF stimulation |  |
| None | 27 (90%) |
| 1 | 1 (3%) |
| 2-3 | 2 (7%) |
| AVM | 18 (60%) |
| CST I | 3 (12%) |
| CST II | 1 (4%) |
| CST III | 7 (27%) |
| CST IV | 14 (53%) |
| CST V | 1 (4%) |

*3 patients missing. CST by Valencia method only available for 26 patients.
